# Supplementary material for: Gating mechanism of hyperpolarization-activated HCN pacemaker channels
Source: Nat Commun. 2020 Mar 17;11:1419. doi: 10.1038/s41467-020-15233-9 (PMC7078272; doi:10.1038/s41467-020-15233-9)
Supplement: Supplementary file 1 — Supplementary Information [file 41467_2020_15233_MOESM1_ESM.pdf]

Gating mechanism of hyperpolarization-activated HCN pacemaker channels

Ramentol *et al.*

Supplementary Figures

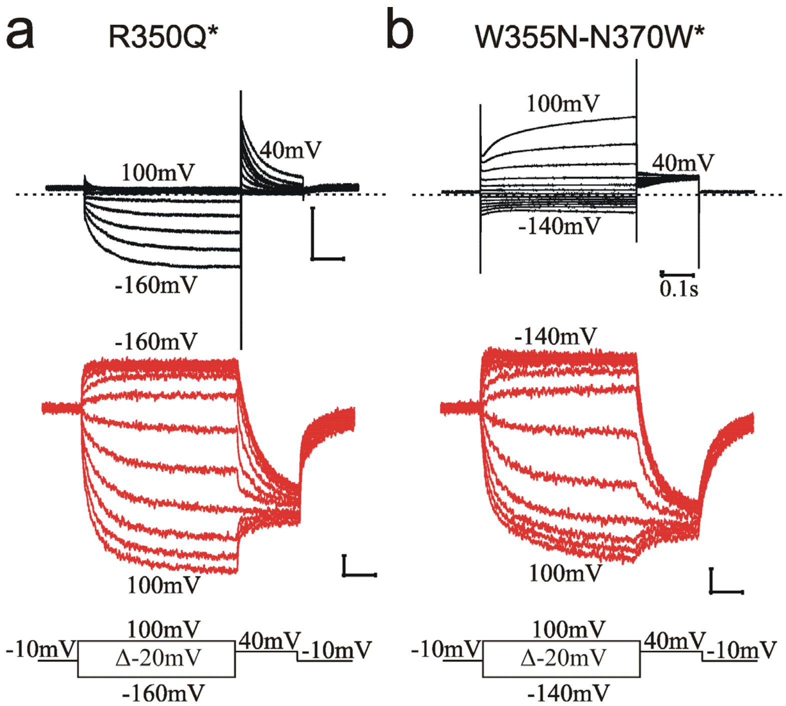

Supplementary Figure 1. Depolarization-activated W355N-N370W* channel and hyperpolarization-activated R350Q* channel have similar S4 movements. **a-b)** Representative currents (black) and fluorescence (red) from **a)** R350Q* and **b)** W355N-N370W* channels. Scale bars: 0.1 s, 1 µA and 0.5% *ΔF/F*. We chose to compare W355N-N370W* with R350* because these channels have a similar $V_{0.5}$ for their *F(V)*s but are opened by opposite voltage polarity.

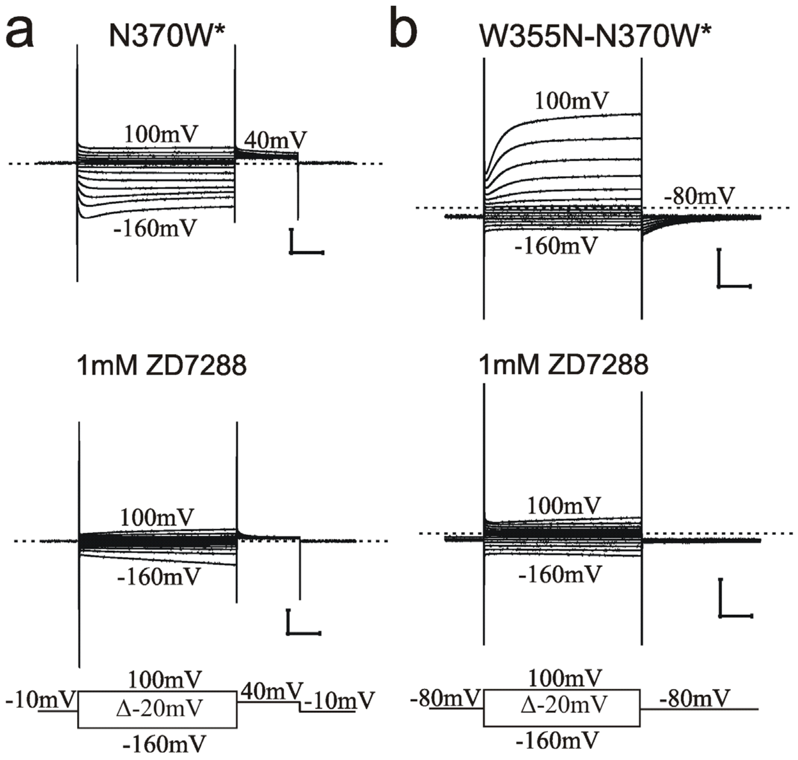

Supplementary Figure 2. Depolarization-activated W355N-N370W* channel is blocked by the specific HCN channel blocker ZD7288. **a-b)** Representative currents from **a)** N370W* and **b)** W355N-N370W* channels before (upper panel) and after (lower panel) application of 1 mM of HCN blocker ZD7288. Scale bars: 0.1 s and 10 μA.

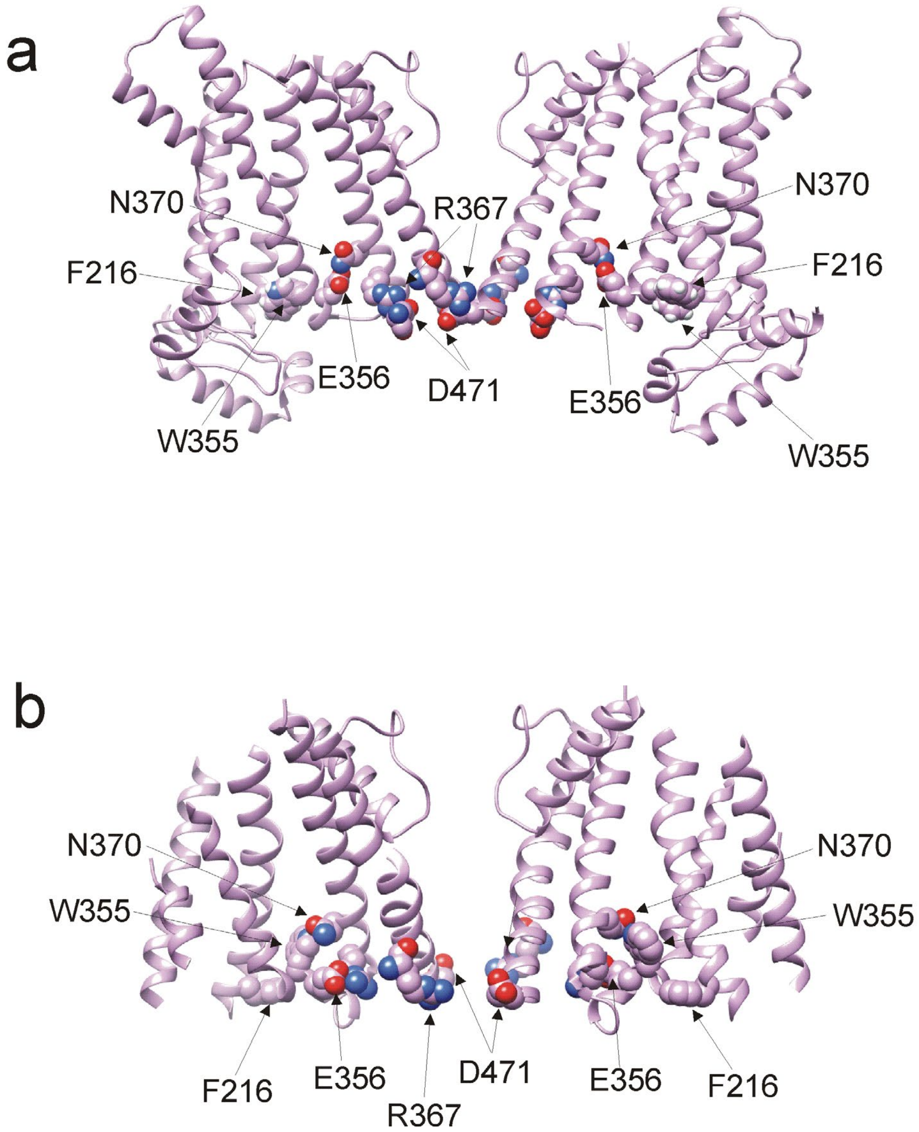

Supplementary Figure 3. Molecular models of spHCN in different states. a) Molecular model of the spHCN channel in the closed state with S4 up based on the cryo-EM structure of hHCN1[1]. Residues mentioned in the text are shown with space-filled side chains as in Supplementary Movie 1. Only two diametrically-opposed subunits are shown for clarity. However, D471 and R367 are shown for all four subunits to show the inter-subunit interaction between these two residues. **b)** Molecular model of the spHCN channel in the open state with S4 up based on the cryo-EM structure of hERG[2]. Residues mentioned in the text are shown with space-filled side chains as in Supplementary Movie 2. Only two diametrically-opposed subunits are shown for clarity. However, D471 is shown for all four subunits to show the separation of R367 and D471 that form an inter-subunit interaction in the closed state.

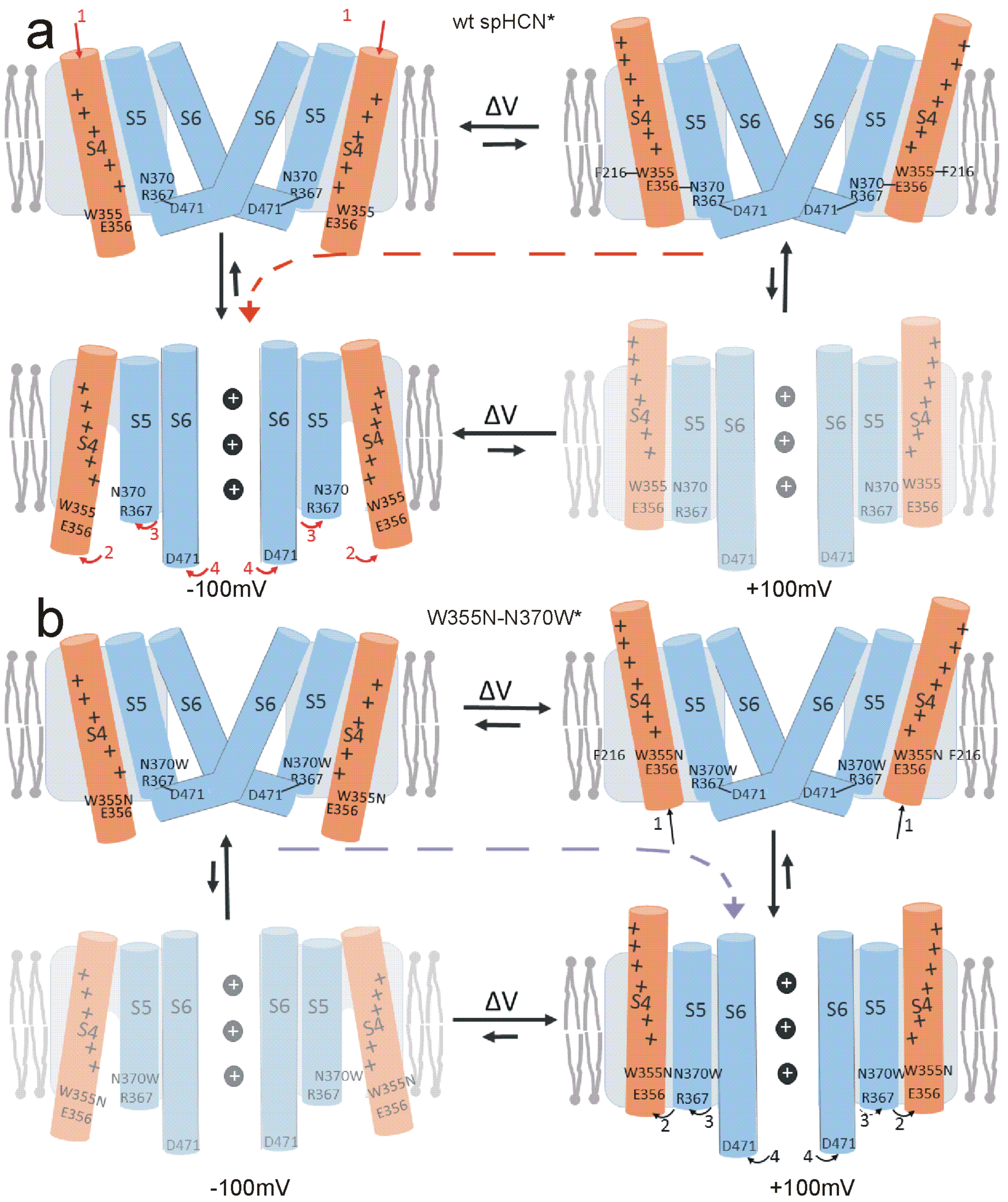

Supplementary Figure 4. Proposed interactions formed during gating of wt and mutant HCN channels.

The four-state allosteric model of spHCN gating is based on that HCN channels have a loose coupling[3], such that the gate can open with S4 in any state (Left: S4 down or Right: S4 up). However, the open probability is much higher with S4 down (vertical arrows). To identify putative residue-residue interactions in the different states, molecular models of spHCN channels in the closed and open state with S4 up were created using cryo-EM structures of the homologous hHCN1[1] and hERG[2] channels, respectively. Molecular models of spHCN channels in the closed and open state with S4 down were created using cryo-EM structures of the homologous hHCN1 and hERG channels, respectively, with the

VSD replaced with the model of the VSD of spHCN with S4 down from the recent paper from Zagotta's group[4].

a) Upper Right) In the wt spHCN* channel in **the closed state with S4 up** (based on hHCN1 cryo-EM structure), W355 interacts with the hydrophobic F216 in S1 by the hydrophobic effect. There is also a hydrogen bond between E356 in S4 and N370 in S5. We hypothesize that these two interactions (W355-F216 and E356-N370) stabilize S4 in its up state and stabilize the closed gate by holding S5 in position to allow an electrostatic interaction between R367 in S5 and D471 in S6, as has previously been suggested to stabilize the closed gate[5]. See Supplementary Figures 3a and 5 for molecular details.

Upper Left) **Closed state with S4 down** (based on the pore of hHCN1 cryo-EM structure and VSD from[4]). We assume that the structure of the closed state of the pore domain is the same independent of the position of S4, only the interactions of the pore domain with the VSD are different with S4 up or S4 down. The downward S4 movement (red 1 arrows) breaks the E356-N370 hydrogen bond and the hydrophobic interaction of W355 with F216 in S1. Instead, W355 is now in a hydrophilic, aqueous environment and N370 is in a hydrophobic environment.

Lower Left) **Open state with S4 down** (based on the pore of hERG cryo-EM structure and VSD from[4]). The initial downward S4 movement (red 1 arrows in Upper Left panel) is followed by a subsequent tilting S4 movement (red 2 arrows) that allows S5 to tilt (red 3 arrows), which destabilizes the R367-D471 interaction. The tilting movement of S4 opens up a water-filled crevice between S4 and S5, exposing N370 to a stabilizing hydrophilic environment. This allows the gate to open at hyperpolarized voltages (red 4 arrows). See Supplementary Movie 1 for a morph between the closed state with S4 up and the open state with S4 down using our molecular models. See Supplementary Movie 1 and Supplementary Figure 5 for molecular details.

Lower Right) **Open state with S4 up** (based on hERG cryo-EM structure). We assume that the structure of the open state of the pore domain is the same independent of the position of S4, only the interactions of the pore domain with the VSD are different with S4 up or S4 down: A morph between closed and open spHCN models with S4 up (Supplementary Movie 2) suggests that the transition from closed to open (when S4 is up) involves a rotation and tilting of lower S4 (black arrow 2) that breaks the W355 interaction with F216 in S1 and the E356-N370 hydrogen bond, a radial translation of lower S5 (black arrow 3) that breaks the R367-D471 interaction, and a tilting and rotation of lower S6 (black arrow 4) that opens the pore. In wt spHCN channels, this opening with S4 up is very infrequent compared to the opening with S4 down (Lower Left) due to the stabilizing interactions in the state with closed gate and S4 up (Upper Right). See Supplementary Movie 2 and Supplementary Figures 3b and 5 for molecular details.

At depolarized voltages (+100 mV), the E356-N370 hydrogen bond, the electrostatic interaction between R367 and D471, and the hydrophobic interaction of W355 with F216 in S1 hold the gate closed when S4 is up in wt HCN channels. So, wt HCN channels are biased to the closed state when S4 is up due to these interactions.

At hyperpolarized voltages (-100 mV), the hydrophilic environment around N370 contributes to biasing the gate open when S4 is down in wt HCN channels.

b) (Lower Left) The W355N-N370W double mutation destabilizes the **open state with S4 down** by introducing a hydrophobic mismatch for N370W in the hydrophilic water-filled crevice.

(Upper Left) Instead, the W355N-N370W mutation promotes channel closing at hyperpolarized potentials and stabilizes the **closed state with S4 down**.

(Upper Right) The W355N-N370W mutation destabilizes the **closed state with S4 up** by removing the W355 hydrophobic interaction with F216 in S1 and the E356-N370 hydrogen bond that normally stabilizes the closed state with S4 up at depolarized potentials.

Lower Right) Instead, the W355N-N370W mutation promotes the o**pen state with S4 up**: A morph between closed and open spHCN models with S4 up (Supplementary Movie 2) suggests that the transition from closed to open (when S4 is up) involves a rotation and tilting of lower S4 (black arrow 2). This transition is promoted in the W355N-N370W mutation because of the absence of an interaction between W355N and F216 in S1 and the absence of an interaction between E356 and N370W. A radial translation of lower S5 (black arrow 3) breaks the R367-D471 interaction and a tilting and rotation of lower S6 (black arrow 4) opens the pore. See Supplementary Movie 2 for a morph between the closed state with S4 up and the open state with S4 up using our molecular models.

At depolarized voltages (+100 mV), N370W cannot make a hydrogen bond with E356 and W355N cannot make a hydrophobic interaction with F216 in S1. Therefore, the electrostatic interaction between R367 and D471 is not enough to keep the channel closed and the gate opens when S4 is up in W355N-N370W channels. So, W355N-N370W channels are biased to the open state when S4 is up due to absence of these interactions.

At hyperpolarized voltages (-100 mV), N370W would be in a hydrophilic environment if the gate opens when S4 is down. Therefore, the W355N-N370W mutation biases the gate closed when S4 is down in W355N-N370W channels.

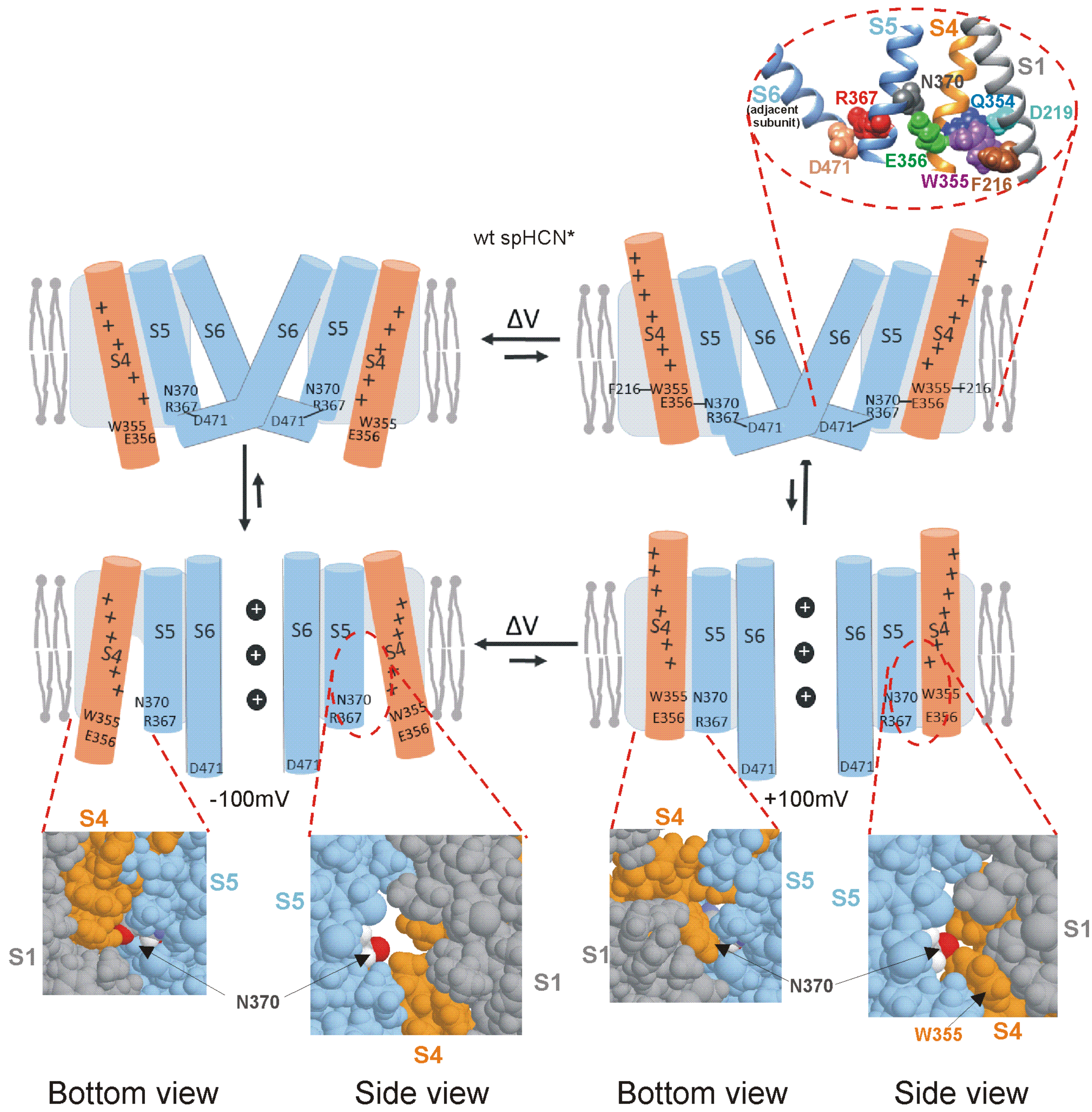

Supplementary Figure 5. Molecular interactions in the different states of spHCN models. Four-state model of spHCN channels. Insets show the molecular interactions and environment of the residues mentioned in the text from the molecular models developed for the different states (See Supplementary Figure 4 and Materials and Methods).

Upper Right) **Closed State with S4 up** (based on the hHCN1 cryo-EM structure): Inset shows side view of the D471-R367, E356-N370, and W355-F216 interactions in the spHCN homology model that we propose stabilize the closed state with S4 up.

Lower left) **Open State with S4 down** (based on the hERG cryo-EM structure of the pore and the VSD structure from Zagotta's group): Inset shows side view and bottom view of N370 in the spHCN homology model. N370 is in this state in a hydrophilic environment with no residue interacting with N370 side chain (side view) and clear access to N370 side chain from the cytosol (bottom view).

Lower Right) **Open State with S4 up** (based on the hERG cryo-EM structure): Inset shows side view and bottom view of N370 in the spHCN homology model. N370 is in this state in a more hydrophobic environment (partly provided by W355) (inset Lower Right) compared to the more hydrophilic environment in the Open State with S4 down (inset Lower Left).

Supplementary Movies.

Supplementary Movie 1. Morph between the closed state of spHCN model with S4 up and the open state of spHCN model with S4 down. The cryo-EM structure of hHCN1 was used for the homology modelling of the closed state of spHCN model with S4 up and the cryo-EM structure of hERG with the VSD replaced for the VSD with S4 down from[4] were used for the homology modelling of the open state of spHCN model with S4 down. A morph between our closed and open HCN models suggests that the transition from closed to open involves a downward movement of S4, that breaks the F216-W355 interaction and E356-N370 hydrogen bond, and a rotation and tilting of lower S4, a radial translation of lower S5, and a tilting and rotation of lower S6 which opens the pore. In the open state with S4 down, N370 is in a hydrophilic water-filled crevice. See Supplementary Figure 3a for identification of residues shown with space-filled side chains.

Supplementary Movie 2. Morph between the closed and open states of spHCN model with S4 up. The cryo-EM structure of HCN1 was used for the homology modelling of the closed state of spHCN model with S4 up and the cryo-EM structure of hERG was used for the homology modelling of the open state of spHCN model with S4 up. A morph between our closed and open HCN models with S4 up suggests that the transition from closed to open (when S4 is up) involves a rotation and tilting of lower S4, a radial translation of lower S5 that breaks the F216-W355 interaction and the E356-N370 hydrogen bond, and a tilting and rotation of lower S6 which opens the pore. See Supplementary Figure 3b for identification of residues shown with space-filled side chains.

Supplementary Methods

Primers used to insert mutations

The forward and reverse primers are identified with a + and -, respectively. The sequence corresponding to the replaced amino acid is shown in uppercase.

R332C+: gccctcaagatactgTGCtttgccaagctcctc

R332C-: gaggagcttggcaaaGCAcagtatcttgagggc

W355N+: catgcggttcgtcagtcaaAATgaacaggccttcaacgtag

W355N–: ctacgttgaaggcctgttcATTttgactgacgaaccgcatg

N370W+: cgtcatccggatctgtTGGctagtgtgtatgatgcttctg

N370W-: cagaagcatcatacacactagCCAacagatccggatgacg

N370G+: cgtcatccggatctgtGGTctagtgtgtatgatgcttc

N370G-: gaagcatcatacacactagACCacagatccggatgacg

N370V+: cgtcatccggatctgtGTTctagtgtgtatgatgcttc

N370V-: gaagcatcatacacactagAACacagatccggatgacg

N370I+: cgtcatccggatctgtATTctagtgtgtatgatgcttctg

N370I-: cagaagcatcatacacactagAATacagatccggatgacg

N370E+: cgtcatccggatctgtGAGctagtgtgtatgatgcttc

N370E-: gaagcatcatacacactagCTCacagatccggatgacg

N370A+: cgtcatccggatctgtGCTctagtgtgtatgatgc

N370A-: gcatcatacacactagAGCacagatccggatgacg

R350Q+: ctgtccaggctcatgCAGttcgtcagtcaatg

R350Q-: cattgactgacgaaCTGcatgagcctggacag

F216A+: gtagtcatttcagaGCTtactgggatctac

F216A-: gtagatcccagtaAGCtctgaaatgactac

ΔQWE+: tcgtcagtcaggccttcaacgtagcc

Δ QWE-: aggcctgactgacgaaccgcatgagc

Supplementary References

1 Lee, C. H. & MacKinnon, R. Structures of the Human HCN1 Hyperpolarization-Activated Channel. *Cell* **168**, 111-120 e111, doi:10.1016/j.cell.2016.12.023 (2017).

2 Wang, W. & MacKinnon, R. Cryo-EM Structure of the Open Human Ether-à-go-go-Related K⁺ Channel hERG. *Cell* **169**, 422-430.e410, doi:10.1016/j.cell.2017.03.048 (2017).

3 Ryu, S. & Yellen, G. Charge movement in gating-locked HCN channels reveals weak coupling of voltage sensors and gate. *The Journal of general physiology* **140**, 469-479, doi:10.1085/jgp.201210850 (2012).

4 Dai, G., Aman, T. K., DiMaio, F. & Zagotta, W. N. The HCN channel voltage sensor undergoes a large downward motion during hyperpolarization. *Nature Structural & Molecular Biology*, doi:10.1038/s41594-019-0259-1 (2019).

5 Decher, N., Chen, J. & Sanguinetti, M. C. Voltage-dependent gating of hyperpolarization-activated, cyclic nucleotide-gated pacemaker channels: molecular coupling between the S4-S5 and C-linkers. *J Biol Chem* **279**, 13859-13865 (2004).
