# Supplementary material for: Gating mechanism of hyperpolarization-activated HCN pacemaker channels
Source: Nat Commun. 2020 Mar 17;11:1419. doi: 10.1038/s41467-020-15233-9 (PMC7078272; doi:10.1038/s41467-020-15233-9)
Supplement: Supplementary file 5 — Description of Additional Supplementary Files [file 41467_2020_15233_MOESM5_ESM.pdf]

Title: Supplementary Movie 1. Morph between the closed state of spHCN model with S4 up and the open state of spHCN model with S4 down.

Description: The cryo-EM structure of hHCN1 was used for the homology modelling of the closed state of spHCN model with S4 up and the cryo-EM structure of hERG with the VSD replaced for the VSD with S4 down from[4] were used for the homology modelling of the open state of spHCN model with S4 down. A morph between our closed and open HCN models suggests that the transition from closed to open involves a downward movement of S4, that breaks the F216-W355 interaction and E356-N370 hydrogen bond, and a rotation and tilting of lower S4, a radial translation of lower S5, and a tilting and rotation of lower S6 which opens the pore. In the open state with S4 down, N370 is in a hydrophilic water-filled crevice. See Supplementary Figure 3a for identification of residues shown with space-filled side chains.

Title: Supplementary Movie 2. Morph between the closed and open states of spHCN model with S4 up.

Description: The cryo-EM structure of HCN1 was used for the homology modelling of the closed state of spHCN model with S4 up and the cryo-EM structure of hERG was used for the homology modelling of the open state of spHCN model with S4 up. A morph between our closed and open HCN models with S4 up suggests that the transition from closed to open (when S4 is up) involves a rotation and tilting of lower S4, a radial translation of lower S5 that breaks the F216-W355 interaction and the E356-N370 hydrogen bond, and a tilting and rotation of lower S6 which opens the pore. See Supplementary Figure 3b for identification of residues shown with space-filled side chains.
